# Identification of a secondary promoter of *CASP8* and its related transcription factor PURα

**DOI:** 10.3892/ijo.2014.2436

**Published:** 2014-05-09

**Authors:** ZHENGWEI LIN, ZHIMIN GUO, YANG XU, XIAOHANG ZHAO

**Affiliations:** 1 State Key Laboratory of Molecular Oncology, Cancer Institute and Hospital, Chinese Academy of Medical Sciences and Peking Union Medical College, Beijing 100021; 2 Center of Basic Medical Science, Navy General Hospital, Beijing 100048, P.R. China

**Keywords:** promoter, *CASP8*, PURα, transcription regulation, esophageal squamous cell carcinoma

## Abstract

Caspase-8 *(CASP8)* is an essential initiator of apoptosis and is associated with many diseases in humans including esophageal squamous cell carcinoma. *CASP8* produces a variety of transcripts, which might perform distinct functions. However, the *cis* and *trans* transcriptional determinants that control *CASP8* expression remain poorly defined. Using a series of luciferase reporter assays, we identified a novel secondary promoter of *CASP8* within chr2: 202,122,236 to 202,123,227 and 25 kb downstream of the previously described *CASP8* promoter. ENCODE ChIP-seq data for this novel promoter region revealed several epigenetic features, including high levels of histone H3 lysine 27 acetylation and lysine 4 methylation, as well as low levels of CpG island methylation. We developed a mass spectrometry based strategy to identify transcription factors that contribute to the function of the secondary promoter. We found that the transcription activator protein PURα is specifically involved in the transcriptional activation of the secondary promoter and may exert its function by forming a complex with E2F-1 and RNA polymerase II. PURα can bind to both DNA and RNA, and functions in the initiation of DNA replication, regulation of transcription. We observed that knockdown of PURα expression decreased the transcriptional activity of the secondary promoter and mRNA expression of *CASP8* isoform G. Although the physiologic roles of this secondary promoter remain unclear, our data may help explain the complexity of *CASP8* transcription and suggest that the various caspase 8 isoforms may have distinct regulations and functions.

## Introduction

The caspase 8 *(CASP8)* gene is located on human chromosome 2q33.1 and plays a vital role in the apoptotic pathway as an initiator caspase (1). *CASP8* is also a crucial factor involved in the defense system against malignant proliferation and tumorigenesis (2–5). When *CASP8* expression is disrupted, RIP3-mediated embryonic lethality is observed in 10.5- to 11.5-day-old embryonic mice, coincident with vascular, cardiac, and hematopoietic defects (6–9). RIP3 plays an essential role in the TNF receptor-1 signaling pathway and can initiate programmed cell necrosis (10). Owing to alternative splicing, *CASP8* produces at least eight different mRNAs (*CASP8* a-h) and shows a very complex pattern of isoform expression (11,12). Different caspase-8 isoforms harbor distinct functional properties, with some even counteracting the apoptosis-initiating effects (12–16). Many studies have explored how *CASP8* regulates apoptosis, but little is known about the transcriptional regulation of *CASP8* and how the widely differing transcripts are produced.

The first *CASP8* promoter was identified in a neuroblastoma cell line, upstream of exon 1 (17–19). Based on the complexity of *CASP8* transcription and the experimental conditions of these studies, however, the possible existence of cryptic or alternate promoters could not be ruled out. Hence, we still know little about the transcription factors responsible for regulating this first promoter, and the transcriptional regulation mechanism of *CASP8* remains to be elucidated.

Owing to the fundamental physiological function of *CASP8* in apoptosis, it is associated with numerous human diseases, especially cancers (20–24). A recent meta-analysis of genome-wide association studies for esophageal squamous cell carcinoma found a susceptibility locus in 2q33.1 encompassing *CASP8* and *ALS2CR12* (25). Therefore, in the present study we closely examined the characteristics of this locus. We identified a second *CASP8* promoter located upstream of the caspase-8 isoform G and developed an effective strategy to identify transcription factors responsible for regulating this newly identified promoter. A comprehensive understanding of the overall transcriptional regulation of *CASP8* will provide insight into the mechanisms that contribute to the etiology of cancers and their responses to treatment.

## Materials and methods

### Cell culture

The esophageal cancer cell line KYSE510 was grown in RPMI-1640 medium (Bioroc, China) supplemented with 10% FBS, 100 U/ml penicillin and 100 *μ*g/ml streptomycin in 5% CO_2_ at 37°C. The human embryonic kidney cell line HEK293 and the esophageal cancer cell line EC0156 were grown in Minimal Essential Medium (Bioroc) supplemented with 10% FBS, 100 U/ml penicillin and 100 *μ*g/ml streptomycin in 5% CO_2_ at 37°C.

### DNA extraction and PCR

Genomic DNA was extracted from KYSE510 cells using the QIAamp DNA Blood Mini kit (Qiagen, Germany). The promoter fragment was synthesized with a TaqDNA polymerase mixture (BioTeke, China). Thermal cycling conditions included activation of the DNA polymerase at 94°C for 5 min followed by 30 cycles at 94°C for 30 sec, 55–65°C for 30 sec, and 72°C for 30 sec. The specific oligonucleotide primers used are shown in Table I.

### Promoter fragment plasmid construction

The amplified promoter fragments were cloned into the pGL3-Basic vector (Promega). The various pGL3-Basic vectors were then digested with *Xho*I and *Hin*dIII (Takara, Japan). The promoter fragments were purified using the Wizard SV Gel and PCR clean up system (Promega), and subsequently ligated into a promoter less pGL3-Basic luciferase reporter vector. To ensure the fidelity of the cloned promoter fragments, all final constructs were sequenced using the vector-specific primers RVprimer 3: 5′-CTAGCAAAATAGGCTGTCCC-3′ and RVprimer 4: 5′-GACGATAGTCATGCCCCGCG-3′.

### Transient transfection and signal detection

For the dual lucif-erase reporter assay, KYSE510 cells were seeded in a 6-well plate at a density of 2×10^5^ cells per well for at least 20 h prior to transfection. The constructed plasmids and the *Renilla* luciferase internal control plasmid (pRL-TK) were transfected into the cells using Lipofectamine (Invitrogen). After 24 h, the cells were treated with the lysis buffer from the Dual-Luciferase Reporter Assay System (Promega). The signals were measured using an automatic microplate reader (Synergy H1, BioTek). For knockdown of the transcriptional activator protein PURα, the cells were transfected with siRNAs (5′-CCACCUAUCGCAACUCCAUTT-3′ and 5′-AUGGAGU UGCGAUAGGUGGTT-3′) for 24 h prior to transfection with the constructed promoter and control plasmids for 24 h. The sequences negative control siRNAs are 5′-UUCUCCGAA CGUGUCACGUTT-3′ and 5′-ACGUGACACGUUCGGA GAATT-3′.

### DNA-protein affinity purification

Nuclear extracts were obtained from KYSE510 cells using the ProteoExtract Subcellular Proteome Extraction kit (Calbiochem, EMD Biosciences Inc., Germany). The protein concentration of the nuclear extract was determined by Bradford method. The primers used to amplify the promoter and non-promoter sequence DNA fragments were labeled with biotin at the 5′ terminus. Streptavidin magnetic beads (Invitrogen) were washed three times with phosphate-buffered saline (PBS) before use. For affinity purification, 3 *μ*g of each biotin-labeled DNA fragment was incubated with 30 *μ*l of the magnetic-bead slurry for 20 min. Unbound DNA fragments were removed, and 500 *μ*g of nuclear protein extracts was added to the streptavidin bead-biotin-labeled DNA fragments and incubated at 4°C overnight. The non-promoter control DNA fragment was used to decrease the abundance of non-specific DNA-binding proteins, such as those that bind histones, in the nuclear extracts. The bead-DNA-protein complex was washed with TBS (50 mM Tris, 300 mM NaCl) three times, and the proteins were eluted using 2% SDS. The eluted proteins were subjected to SDS-PAGE, and visualized by silver staining. Protein bands were excised and identified by in-gel trypsin digestion with subsequent analysis by MS (Q Exactive Orbitrap, Thermo Scientific). Mascot version 2.3.01 (Matrix Science Inc.) was used to analyze the data and search the databases.

### Western blot analysis

The elution products from affinity purification or cell lysates were denatured in SDS-PAGE sample buffer containing 0.5 M Tris-HCl (pH 6.8), 2% SDS, 10% DTT, 10% glycerol and 0.01% bromophenol blue, boiled for 5 min, and then analyzed by SDS-PAGE followed by transfer to a PVDF membrane (Millipore, Germany). Each membrane was blocked for 1 h in PBS containing 10% nonfat milk. After blocking, each membrane was incubated overnight with rabbit polyclonal anti-E2F-1, mouse monoclonal anti-PURα, or rabbit polyclonal anti-RNA polymerase II (Pol II) (Santa Cruz Biotechnology Inc.). After washing with TBS containing 20% (w/v) Tween-20, each membrane was incubated with horseradish peroxidase-conjugated secondary antibody for 2 h and visualized with the SuperSignal West Femto Maximum Sensitivity Substrate (Thermo Fisher Scientific).

### Immunoprecipitation assay

KYSE510 cells cultured in 10-cm dishes to 90% confluency were washed with ice-cold PBS and lysed for 30 min on ice in lysis buffer containing 1% (w/v) Triton X-100, 0.15 M NaCl, and 30 mM Tris-HCl (pH 7.5) with protease inhibitors (Roche, Germany). Lysates were sonicated and centrifuged at 10,000 × g at 4°C for 15 min. The protein concentration was determined by Bradford assay. For immuno precipitation, 1 mg of the resulting extract was incubated at 4°C overnight with anti-PURα or anti-E2F-1 and Dynabeads Protein G (Invitrogen). Immunoprecipitates were washed three times with lysis buffer and the beads were directly boiled in 1% SDS-PAGE loading buffer.

### Immunofluorescence under confocal microscopy

For immuno fluorescence, KYSE510 cells were fixed in 10% (w/v) paraformaldehyde on poly-L-lysine-coated slides for 30 min at room temperature and washed three times with PBS (pH 7.4). The cells were blocked with PBS (pH 7.5) supplemented with 1% (w/v) BSA and 0.1% (w/v) Triton X-100 for 30 min at room temperature. Washed cells were incubated for 30 min at room temperature with primary rabbit anti-human E2F-1 and mouse anti-human PURα (Santa Cruz Biotechnology Inc.). Then the cells were incubated in the dark for 60 min with Alexa Fluor 488-conjugated goat anti-rabbit and Alexa Fluor 594-conjugated goat anti-mouse (Life Technologies). Cells were stained with DAPI and examined using fluorescence confocal microscopy (Leica Tcs SP2, Germany).

### Quantitative RT-PCR

The total mRNA was extracted using TRIzol (Invitrogen). After the quality control was examined, mRNA was transformed to cDNA by reverse transcriptase kit (Tiangen). The quantitative PCR was completed using SYBR Green Master (ROX) (Roche), and the system included SYBR Green Master (ROX) (2X) 10.0 *μ*l, PCR forward primer (10 *μ*M) 0.6 *μ*l, PCR reverse primer (10 *μ*M) 0.6 *μ*l, Template cDNA 2.0 *μ*l, ddH_2_O ≤20.0 *μ*l. The reaction was conducted under ABI PRISM^®^ 7500. The quality control and Ct values of the reaction were analyzed using SDS software.

### ENCODE database analysis

**Figure 1.**
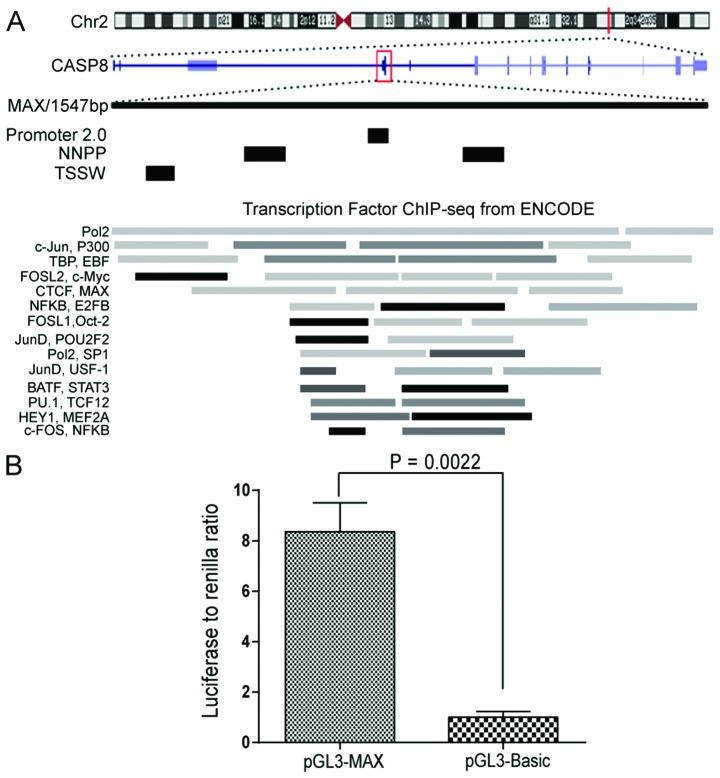
Identification of the MAX fragment on chromosome 2 that shows transcriptional activity. (A) The black boxes in the upper portion represent the hypothetical promoter regions predicted by the programs Promoter 2.0 (64), NNPP (65), and TSSW (66). The bars in the lower portion indicate potential binding regions for the indicated transcription factors in the fragment of interest based on the ENCODE ChIP-seq database. The degree of shading of the bars indicates the signal intensity. (B) Luciferase reporter assay in KYSE510 cells. The MAX fragment (1547 bp) was introduced into the promoter-deficit pGL3-Basic vector and transiently transfected into KYSE510 cells. The original transcription activities of the fragment were calculated as the ratio to the intensity value of pRL-TK internal control vector. To compare the ratios to that of the promoter-deficit pGL3-Basic vector, we normalized all the ratios by that of pGL3-Basic vector. Values represent the mean ± standard deviation of three independent experiments. The P-value was obtained by t-test of two independent samples.

ENCODE is a DNA elements encyclopaedia in human cell lines (26). We mainly used the ChIP-seq, histone methylation and DNA methylation data. The human cell lines represented in Fig. 1 from ENCODE include GM12878 (lymphoblastoid cells), H1-hESC (embryonic stem cells), K562 (bone marrow), HeLa S3 (cervix adenocarcinoma epithelial cells), HEP G2 (hepatocellular carcinoma cells), HUVEC (umbilical vein endothelial cells), A549 (lung carcinoma cells), IMR90 (lung fibroblasts), MCF-7 (breast cancer epithelial cells), and HESC (embryonic stem cells).

### Statistical analysis

The data presented are the mean ± standard deviation. To examine differences between two groups in the luciferase reporter assay, t-tests were applied using SPSS version 17.0 (IBM software). Mann-Whitney U test was used to compare the mRNA expression of two groups in the RT-PCR experiments. P-values <0.05 were considered statistically significant.

## Results

### A fragment within CASP8 on chromosome 2 shows transcriptional activity

Analysis of data from ChIP coupled with deep sequencing (ChIP-seq) in the Human Encyclopedia of DNA elements (ENCODE) database revealed a region on human chromosome 2q33.1 that contained binding sites for numerous transcription factors and thus might be involved in transcriptional regulation of *CASP8*. This region is located within chr2: 202,122,236 to 202,123,227 and 25 kb downstream of the previously described *CASP8* promoter. Transcript of caspase-8 isoform G is adjacent to this region. To examine whether this region has promoter characteristics, we analyzed this fragment (1547 bp, termed MAX) using three promoter prediction programs; four potential promoter sequences were identified (Fig. 1A). To confirm that the fragment is transcriptionally active, we introduced MAX into a promoter-deficit luciferase reporter vector (pGL3-Basic). When transfected into KYSE510 cells, the pGL3-MAX construct resulted in considerably higher luciferase activity than the pGL3-Basic vector alone (P=0.0022, Fig. 1B).

### Core promoter of the fragment is restricted to the region with transcription-related epigenetic modifications

**Figure 2.**
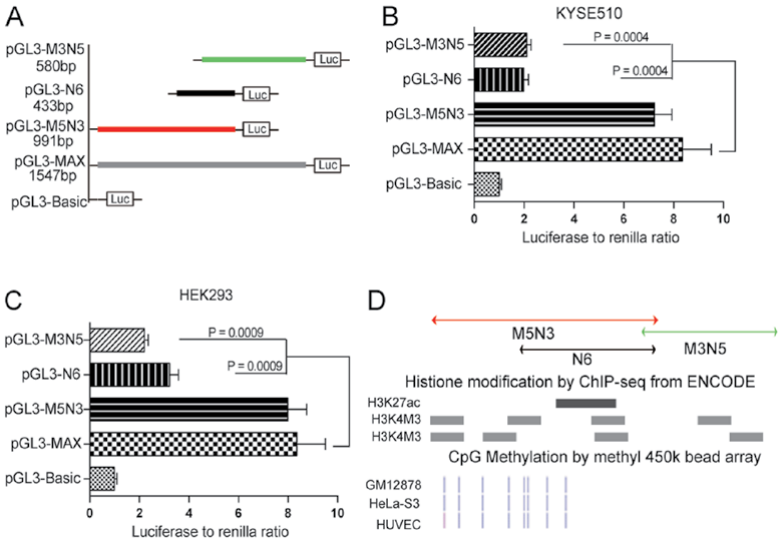
Luciferase reporter assay and transcription-related epigenetic modifications of different truncated MAX fragments. (A) Schematic illustration of the truncated MAX constructs. The position of the box represents the fragment location relative to the full-length MAX. (B) Luciferase reporter activity of the MAX constructs in KYSE510 cells. (C) Luciferase reporter activity of the MAX constructs in HEK293 cells. (D) Histone modification and CpG methylation in the regions covered by the three truncated MAX fragments observed in several cell lines in the ENCODE database. The boxes (black and grey) show where the indicated promoter-associated modifications (acetylation of lysine 27 in histone H3 or trimethylation of lysine 4 in histone H3) occur in the given cell lines (H1-hESC, GM12878 and K562). The color-coded vertical lines represent CpG islands, with bright blue indicating unmethylated and purple indicating partially methylated.

To identify the core promoter region within the 1547-bp MAX fragment, we first constructed reporter vectors containing three different imbricating truncations of MAX that were transfected into KYSE510 cells for the luciferase activity assay (Fig. 2A). The construct containing the 5′-terminus of MAX (pGL3-M5N3) retained luciferase activity comparable to MAX, whereas constructs containing 3′-terminal fragments (pGL3-N6 and pGL3-M3N5) led to statistically significant decreases in transcriptional activity (76.2%, P=0.0004, and 74.8%, P=0.0004, respectively; Fig. 2B). These results suggested that the region contained in pGL3-M5N3 encompassed the core of this novel *CASP8* promoter. Data obtained with KYSE510 cells was validated in HEK293 cells, in which we observed similar results for pGL3-M5N3, pGL3-N6 and pGL3-M3N5 (Fig. 2C).

We also examined potential epigenetic modifications of these three truncated fragments by analyzing these regions in the ENCODE database. Histone modifications, including acetylation of lysine 27 in histone H3 and trimethylation of lysine 4 in histone H3, are indicative of actively transcribed promoters. There was significant enrichment of these two kinds of histone modifications within fragment M5N3 compared with fragments M3N5 and N6 (Fig. 2D). CpG methylation status is also related to the degree of transcriptional activation of a DNA region (27). All of the CpG sites in M3N5 were methylated according to the ENCODE database, whereas the M5N3 region contained many unmethylated or partially methylated CpG sites, which also suggested that fragment M5N3 was more transcriptionally active than M3N5 and N6 (Fig. 2D). We therefore concluded that fragment M5N3 encompasses the core sequence of this novel *CASP8* promoter.

**Figure 3.**
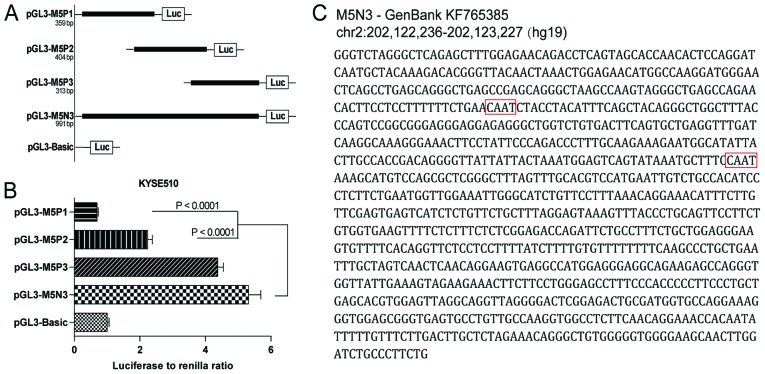
Analysis of the core promoter region of *CASP8*. (A) Schematic illustration of the truncated constructs derived from M5N3. The position of the black boxes represents the fragment location relative to the full M5N3. (B) Luciferase reporter activity of the M5N3 fragments in KYSE510 cells. (C) Genomic sequence of the core promoter region (M5N3) with the putative CAAT box outlined in red.

The pGL3-M5N3 construct (991 bp) only decreased the luciferase activity in KYSE510 cells by 13.7% relative to the full MAX fragment (Fig. 2B). We continued to explore the parts of this fragment that were most vital for maintaining transcriptional function. Three imbricating truncations of M5N3 were introduced into pGL3-Basic (pGL3-M5P1, 359 bp; pGL3-M5P2, 404 bp; pGL3-M5P3, 313 bp) and transfected into KYSE510 cells (Fig. 3A). Comparing the activities of these three constructs to that of pGL3-M5N3, we found that pGL3-M5P1 and pGL3-M5P2 decreased the transcriptional activity significantly (P<0.0001), whereas pGL3-M5P3 retained comparable activity (Fig. 3B). The M5P3 sequence (Fig. 3C), which likely contains the essential core of this newly identified promoter, has been submitted to GenBank under accession number KF765385.

### DNA-protein affinity purification combined with LC-MS/MS identifies PURα as a specific transcription factor for the newly identified promoter

**Figure 4.**
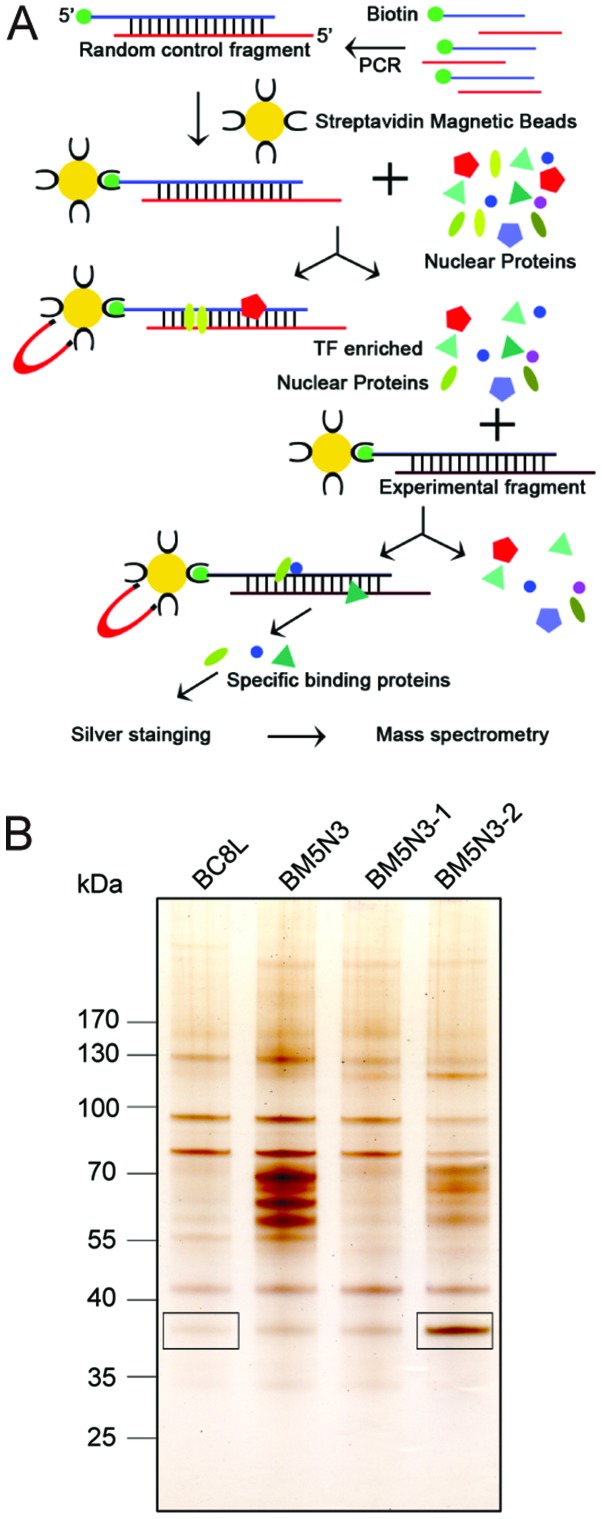
Isolation and identification of specific promoter fragment-binding proteins. (A) A flow chart of the separation and identification process. First, a biotin-labeled non-promoter control fragment was incubated with nuclear proteins to decrease the abundance of non-specific DNA-binding proteins, and the bound proteins were removed with streptavidin-coupled magnetic beads. The transcription factor-enriched nuclear proteins were then incubated with the experimental fragments to obtain the specific binding proteins. The released proteins were then electrophoresed and silver stained to compare control and experimental fragment-bound fractions, and shotgun MS was used to identify the proteins in the experimental fragment-specific bands. (B) Silver-stained gel of the elution products from the control (BC8L) and experimental (BM5N3) fragments. The nuclear proteins in the BM5N3 lane were not pre-incubated with the control fragment, whereas the protein extract in the BM5N3-1 lane was pre-incubated one time and the protein extract in the BM5N3-2 lane was pre-incubated two times. The band in the black rectangle went into the shotgun MS identification step.

To identify which transcription factors contribute to the activity of new promoter, we developed a strategy to separate, enrich and identify the nuclear proteins that specifically bind to fragment M5N3 (Fig. 4A). The biotin-labeled fragment (BM5N3) was conjugated to streptavidin-coupled magnetic beads and then incubated with nuclear proteins extracted from KYSE510 cells. To eliminate non-specific DNA-binding proteins, such as those binding histones, we pre-incubated the nuclear proteins with a non-promoter control fragment from a conserved exon of most *CASP8* transcripts in which ENCODE ChIP-seq data suggested there are no potential transcription factor-binding sites (BC8L; Fig. 4B). The bound proteins were eluted and examined by SDS-PAGE. Silver staining of the gels revealed several bands that were enriched in the BM5N3 elutions compared with the BC8L elution. Most notably, when the nuclear extracts were pre-incubated twice with the control fragment (BM5N3-2), a greatly enriched band was evident in the region in which most transcription factors are distributed (35–55 kDa; Fig. 4B).

To identify the proteins in the specific enriched bands in the promoter fragment elutions (Fig. 4B), we extracted the bands and performed LC-MS/MS. Based on the MS/MS spectra of the peptides, we identified proteins using Mascot software. After excluding proteins that were also present in the corresponding control bands, we retained the 12 promoter-specific proteins listed in Table II. The selected proteins were analyzed in terms of three parameters, peptide score, molecular mass, and subcellular localization. We excluded four proteins (KLP6, MUCL1, GNAS and FBN3) based on the fact that their actual known molecular mass fell outside the 35- to 55-kDa gel region that we extracted. Two other proteins (PDHA1 and CLEC14A) were excluded because of their low peptide scores. Of the remaining six proteins, the transcriptional activator protein PURα showed the appropriate subcellular localization and known function and thus was chosen as the candidate transcription factor to be validated.

**Figure 5.**
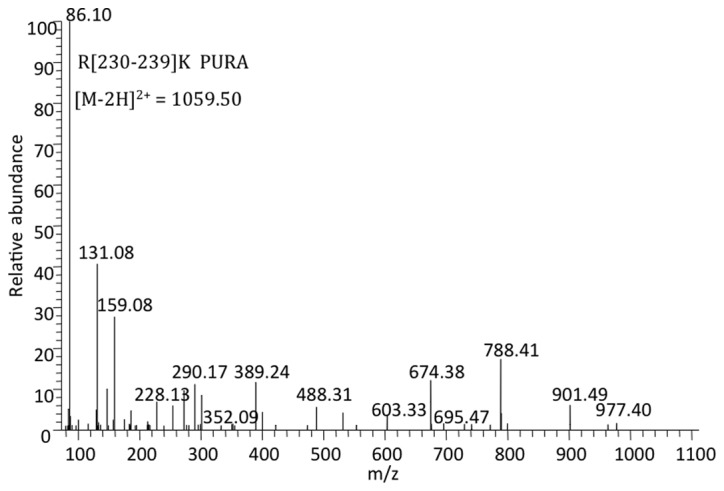
The Identification of banding proteins by LC-MS/MS. The MS data and peptide score distribution were evaluated using Mascot. The figure shows MS/MS spectrum of the unique peptide (PURα).

The MS/MS spectrum showed a peptide with mass 1059.50 Da reconstituted from the doubly charged ion of 530.76 m/z. The sequence obtained from this spectrum revealed that the peptide originated from arginine 230 to lysine 239 [R(230–239)K] of PURα [National Center for Biotechnology Information (NCBI) gene identifier 5813] and was unique to PURα (Fig. 5). Mascot software calculated the probability (P-value) of the identified peptide being a random event and transformed the P-value to a peptide score (the peptide score was 20 when P=0.05). The peptide score of the unique peptide from PURα was 41 (P=0.00074), indicating the reliability of the result.

### PURα may exert its function by interacting with E2F-1

**Figure 6.**
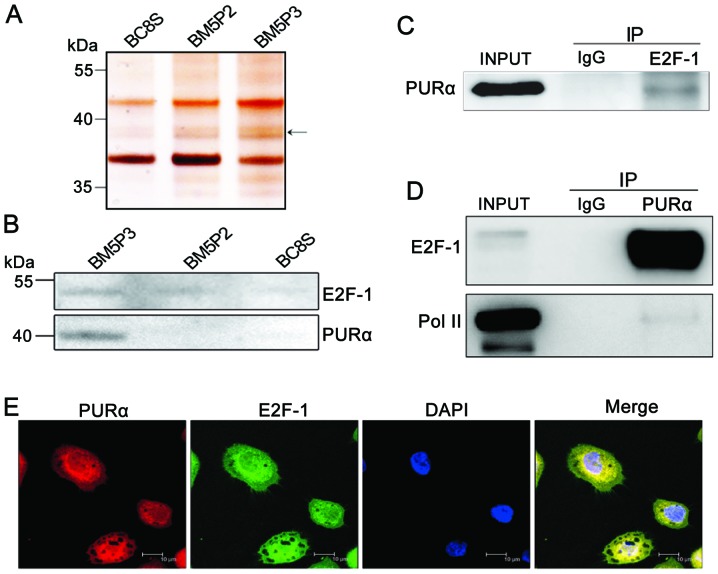
PURα interacts with E2F-1. (A) Silver-stained gel of the elution products from an affinity pull-down assay with the core promoter region. BM5P3 also pulled down a 40-kDa protein. (B) Western blot analysis of the elution products with PURα and E2F-1 antibodies. (C) Protein immunoprecipitation (IP) assay using anti-E2F-1 or non-specific IgG as a control. Immunoprecipitates were analyzed for the presence of PURα. (D) Protein immunoprecipitation assay using anti-PURα or non-specific IgG as a control. Immunoprecipitates were analyzed for the presence of E2F-1 and RNA polymerase II (Pol II). E) Confocal micrographs of PURα and E2F-1 immunofluorescence in KYSE510 cells. Nuclei were stained with DAPI. The merge image shows colocalization of PURα and E2F-1.

To validate the MS/MS results, we first repeated the DNA-protein affinity purification using just the core promoter region M5P3. Silver staining of the electrophoresed elution products showed a significantly enriched band at ∼40 kDa, which was identical to the band isolated with the longer M5N3 fragment (Fig. 6A). Western blotting of the eluted products confirmed the presence of PURα. We also examined E2F-1 in the western blotting (Fig. 6B) because PURα and E2F family members are known to inter-regulate (28). To determine if PURα and E2F-1 physically interact, we performed reciprocal immunoprecipitation assays. The results showed that PURα and E2F-1 directly or indirectly interacted (Fig. 6C and D). Pol II is responsible for synthesizing messenger RNA in eukaryotes and is an essential part of the transcriptional machinery (29,30). A Pol II signal was also detected in the PURα immunoprecipitates (Fig. 6D), suggesting that PURα and E2F-1 are likely components of the transcriptional complex. Immunohistochemistry with PURα and E2F-1 antibodies and confocal microscopy confirmed the colocalization of these proteins in the nucleus of KYSE510 cells (Fig. 6E).

### Knockdown of PURα attenuates the transcriptional activity of the novel promoter and the mRNA expression of CASP8 isoform G

**Figure 7.**
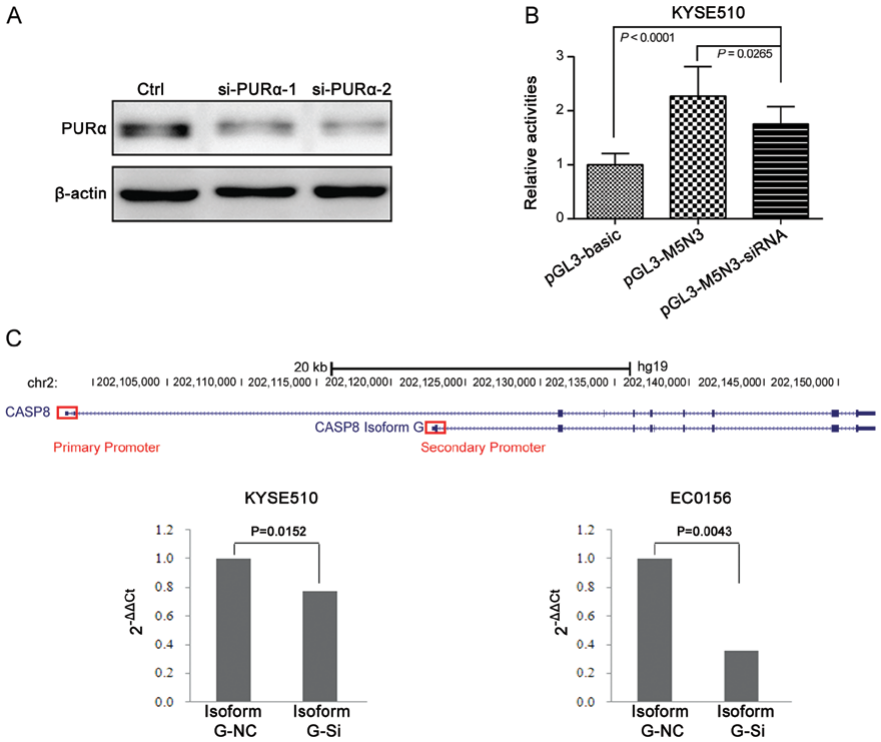
The novel promoter activity and mRNA expression of *CASP8* isoform G require PURα expression. (A) After transfection with siRNA, the knockdown of PURα was confirmed by western blotting. The Ctrl lane is the negative control group transfected with random control siRNAs. (B) The promoter activity of the construct pGL3-M5N3 was examined in KYSE510 cells transfected with negative control siRNA or PURα-specific siRNA by the luciferase reporter assay. (C) The upper panel is the relative position of primary promoter and secondary promoter within gene *CASP8*. The two transcripts stand for two kinds transcripts of *CASP8* based on their transcription starting sites. The rectangles encompass the two promoter regions. The lower panel shows the quantitative RT-PCR results of the KYSE510 and EC0156 cell lines, knocking down PURα by siRNA (Si) or not (NC). The y-axis are values of 2^−ΔΔCt^, and ΔCt equals the gene average Ct minus the internal control *(GAPDH)* average Ct.

To evaluate the function of PURα and its contribution to the transcriptional machinery, we knocked down PURα in KYSE510 cells using siRNA-mediated RNAi and then transfected the pGL3-M5N3 construction to these cells and measured promoter activity. Western blotting confirmed that the siRNA decreased PURα protein expression (Fig. 7A). The pGL3-M5N3 promoter activity was still higher than the promoter-deficient pGL3-Basic plasmid in the PURα knockdown strain (P<0.0001) but was significantly lower than for pGL3-M5N3 without PURα knockdown (P=0.0265; Fig. 7B). Based on the results of transcriptional activity, we continued to test whether PURα could influence the mRNA expression of gene *CASP8*. The secondary promoter locates surround the 5′-UTR of isoform G, so we designed primers specific to isoform G. We observed significant down regulation of mRNA expression of isoform G in KYSE510 cells after knowing down PURα (P=0.0152; Fig. 7C). We validated this result in another esophageal cell line (EC0156; P=0.0043; Fig. 7C). These results point to PURα as responsible for the transcriptional activity of the secondary promoter and it is able to promote the mRNA expression of isoform G.

## Discussion

Transcriptional regulation is a complex and dynamic process involving concerted modulation of transcription initiation, alternative splicing and post-transcriptional modifications (31–33). Complex transcriptional units can produce multiple mature mRNAs by a variety of mechanisms, including alternative splicing or use of alternative promoters or alternative start sites around a single promoter (34,35). Mammalian promoters have been identified at various unexpected positions in the genome, such as in intergenic regions far from known genes, in the 3′-UTRs of known protein-coding genes, in coding exons, and in introns (36–38). Many human genes have secondary or alternative promoters (39–43).

At least eight different *CASP8* transcripts have been identified. This variety of transcripts can be explained, in part, by transcription from the known promoter of *CASP8* (17–19). Of note, a six-nucleotide insertion-deletion polymorphismin this promoter may be associated with susceptibility to multiple cancers and can influence expression of certain *CASP8* isoforms (44). However, results are contradictory about the association of this polymorphism with disease (45–54). We believe that this contradiction stems, in part, from the existence of the second promoter that we identified for *CASP8*, which suggests more complex transcriptional regulation than previously thought. The ENCODE project aims to identify all functional elements in the human genome sequence (26,55). Based on this database, some new features and mechanisms of transcriptional systems have been characterized at the overall genome level (56,57). To further uncover the regulatory mechanisms underlying individual genes, we suggest analysis of the ENCODE data for specific region, as was done in this study. The ENCODE database provides an excellent platform to identify potential unknown DNA elements, like secondary promoters.

Identification of transcription factors associated with a specific gene has often relied on analysis of binding sites for known candidate factors and on ChIP-seq techniques, neither of which can identify novel factors (58). In this study, we developed a strategy that incorporated MS to identify proteins that specifically bind a selected DNA fragment. This strategy could easily be applied to the identification of binding proteins for many other DNA elements and thus could greatly expand the depth of research on transcriptional regulation.

PURα is a member of the PUR family of proteins, it can bind to both DNA (either single- or double-stranded) and RNA, and functions in the initiation of DNA replication, regulation of transcription and mRNA translation (59). PURα is associated with many types of neoplasias and brain development (60). As a single-stranded nucleic acid-binding protein, PURα has DNA helix-destabilizing activity, which is consistent with the requirement for duplex DNA unwinding during initiation of transcription and replication (61). The fact that PURα knockdown decreased the activity of the secondary promoter fragment suggests that this protein is directly involved in *CASP8* transcription. The RT-PCR results further confirmed that PURα was responsible for the mRNA expression of *CASP8*, especially certain transcripts such as isoform G. Isoform G is the longest isoform of caspase-8 (538 amino acids), also known as procaspase-8L. Apart from the well known apoptosis role, caspase-8 also has some nonapoptotic functions such as regulation of proliferation and differentiation of B cells and NK cells (62,63), and these functions might be exerted by a certain isoform. PURα interacts with many transcription factors, including E2F-1 (28). We found E2F-1 at the second *CASP8* promoter and confirmed the physical interaction between PURα and E2F-1 and also revealed a relationship between PURα and Pol II.

In summary, we identified a secondary promoter of *CASP8* in the 5′-UTR and exon 1 of isoform G. Through affinity purification combined with MS, we identified PURα as a promoter-specific transcription factors that appears to function together with E2F-1. The presence of this functional secondary promoter in *CASP8* suggests a complex pattern of gene regulation and may also explain some of the contradictory results obtained in previous studies of *CASP8*. Further research on the complicated regulation mechanism of *CASP8* will provide a greater understanding of programmed cell death.

## Figures and Tables

**Table I.** Primers used in PCR and plasmids construction.

Name	Sequence (5′-3′)
Max-forward	5′-GGGTCTAGGGCTCAGAGCTT-3′
Max-reverse	5′-CAGTCACCTCTGGAGGCATT-3′
M5N3-forward	5′-GGGTCTAGGGCTCAGAGCTT-3′
M5N3-reverse	5′-ACTTGGATCTGCCCTTCTG-3′
N6-forward	5′-CCTGCAGTTCCTTCTGTGGT-3′
N6-reverse	5′-ACTTGGATCTGCCCTTCTG-3′
M3N5-forward	5′-CCTGCAGTTCCTTCTGTGGT-3′
M3N5-reverse	5′-AATGCCTCCAGAGGTGACTG-3′
M5P1-forward	5′-GGGTCTAGGGCTCAGAGCTT-3′
M5P1-reverse	5′-CCCTGTCGGTGGCAAGTAAT-3′
M5P2-forward	5′-GCCACCGACAGGGGTTATTA-3′
M5P2-reverse	5′-GCCACCGACAGGGGTTATTA-3′
M5P3-forward	5′-CAAGCCCTGCTGAATTTGCT-3′
M5P3-reverse	5′-CAGAAGGGCAGATCCAAGT-3′
C8L-forward	5′-TCAGGCTTGTCAGGGGGAT-3′
C8L-reverse	5′-CTGCAGCTACTCCCACCTTC-3′
Isoform G-forward	5′-CACAGGTTCTCCTCCTTTTATCTT-3′
Isoform G-reverse	5′-TTCAATAACCACCCTGGCTCTTC-3′
GAPDH-forward	5′-ACAGCAACAGGGTGGTGGAC-3′
GAPDH-reverse	5′-TTTGAGGGTGCAGCGAACTT-3′
Bio-Max-forward	5′-biotin-GGGTCTAGGGCTCAGAGCTT-3′
Bio-N6-forward	5′-biotin-CCTGCAGTTCCTTCTGTGGT-3′
Bio-M5P3-forward	5′-biotin-CAAGCCCTGCTGAATTTGCT-3′
Bio-M5P2-forward	5′-biotin-GCCACCGACAGGGGTTATTA-3′
Bio-C8L-forward	5′-biotin-TCAGGCTTGTCAGGGGGATA-3′

**Table II.** Proteins identified by LC-MS/MS specific to promoter fragment.

Uniprot	Score	Mass	Protein	Gene
P25311	68	34237	Zinc-α-2-glycoprotein	*AZGP1*
Q00577	55	34889	Transcriptional activator protein Pur-α	*PURA*
Q9P0J7	36	41919	E3 ubiquitin-protein ligase KCMF1	*KCMF1*
P15328	35	29799	Folate receptor α	*FOLR1*
P04406	29	36030	Glyceraldehyde-3-phosphate dehydrogenase	*GAPDH*
Q8NBX0	27	47121	Saccharopine dehydrogenase-like oxidoreductase	*SCCPDH*
B7ZC32	25	108185	Kinesin-like protein KLP6	*KLP6*
Q96DR8	24	9034	Mucin-like protein 1	*MUCL1*
Q5JWF2	22	110956	Guanine nucleotide-binding protein G(s) subunit α	*GNAS*
P08559	22	43268	Pyruvate dehydrogenase E1 component subunit α	*PDHA1*
Q86T13	17	51603	C-type lectin domain family 14 member A	*CLEC14A*
Q75N90	15	300149	Fibrillin-3	*FBN3*
